# Glycyrrhetinic acid might increase the nephrotoxicity of bakuchiol by inhibiting cytochrome P450 isoenzymes

**DOI:** 10.7717/peerj.2723

**Published:** 2016-11-22

**Authors:** Aifang Li, Nana Ma, Zijing Zhao, Mei Yuan, Hua Li, Qi Wang

**Affiliations:** 1 Department of Toxicology, School of Public Health, Peking University, Beijing, China; 2 Beijing Institute of Pharmacology and Toxicology, Beijing, China; 3 State Key Laboratory of Toxicology and Medical Countermeasures, Beijing, China; 4 Beijing Key Laboratory of Toxicological Research and Risk Assessment for Food Safety, Beijing, China; 5 Key Laboratory of State Administration of Traditional Chinese Medicine (TCM) for Compatibility Toxicology, Beijing, China

**Keywords:** Cytochrome p450, Glycyrrhetinic acid, Toxicokinetics, Bakuchiol, Nephrotoxicity

## Abstract

**Background:**

Licorice, a popular traditional Chinese medicine (TCM), is widely used to moderate the effects (detoxification) of other herbs in TCM and often combined with *Fructus Psoraleae*. However, the classical TCM book states that *Fructus Psoraleae* is incompatible with licorice; the mechanism underlying this incompatibility has not been identified. Glycyrrhetinic acid (GA), the active metabolite of licorice, may increase the toxicity of bakuchiol (BAK), the main chemical ingredient in *Psoralea corylifolia*, by inhibiting its detoxification enzymes CYP450s.

**Methods:**

The effect of concomitant GA administration on BAK-induced nephrotoxicity was investigated, and the metabolic interaction between BAK and GA was further studied in vitro and in vivo. The cytotoxicity was assessed using an MTT assay in a co-culture model of HK-2 cell and human liver microsomes (HLMs). The effect of GA on the metabolism of BAK, and on the activities of CYP isoforms were investigated in HLMs. The toxicokinetics and tissue exposure of BAK as well as the renal and hepatic functional markers were measured after the administration of a single oral dose in rats.

**Results:**

*In vitro* studies showed that the metabolic detoxification of BAK was significantly reduced by GA, and BAK was toxic to HK-2 cells, as indicated by 25∼40% decreases in viability when combined with GA. Further investigation revealed that GA significantly inhibited the metabolism of BAK in HLMs in a dose-dependent manner. GA strongly inhibits CYP3A4 and weakly inhibits CYP2C9 and CYP1A2; these CYP isoforms are involved in the metabolism of BAK. *In vivo* experiment found that a single oral dose of BAK combined with GA or in the presence of 1-aminobenzotriazole (ABT), altered the toxicokinetics of BAK in rats, increased the internal exposure, suppressed the elimination of BAK prototype, and therefore may have enhanced the renal toxicity.

**Conclusion:**

The present study demonstrated that GA inhibits CYP isoforms and subsequently may increase the nephrotoxicity of BAK, which underlie one of the possible mechanisms responsible for the incompatibility of Licorice with *Fructus Psoraleae*.

## Introduction

*Fructus Psoraleae* (Buguzhi), dry fruit of* Psoralea* *corylifolia* L. or *Psoralea cordata* (Thunb.) Salter, is widely used in Traditional Chinese Medicine (TCM) as well as in Ayurvedic medicine (Chopra, Dhingra & Dhar, 2013). Bakuchiol (BAK) is the main chemical constituent of *Fructus Psoraleae*, constituting about 1.12–5.14% (Qiao et al., 2007). BAK was found to exert hepatoprotective (Cho et al., 2001; Park et al., 2005), antioxidative (Adhikari et al., 2003), antitumor (Chen et al., 2010), and anti-aging (Chaudhuri & Bojanowski, 2014) effects. Moreover, high-dose BAK is toxic to mouse kidneys (Zhang, 1981) and cytotoxic to human renal tubular epithelial cells (Human Kidney-2, HK-2) (Iwamura et al., 1989; Jiang et al., 2010). We previously found (Jiao et al., 2011; Li et al., 2012) that BAK is extensively metabolized in human liver microsomes (HLMs). A group of CYP isozymes, including CYP2C9, CYP2C19 and CYP3A4, are involved in BAK metabolism. The metabolism of BAK is mediated by CYP isozymes via a detoxification pathway, and 1-aminobenzotriazole (ABT), a broad spectrum inhibitor of CYP, reversed the CYP-mediated detoxification of BAK in HLMs (Li et al., 2012) .

Licorice (Liguorice), a popular TCM has been used in clinical practice (Wang et al., 2013) and a food sweetener in many countries (Isbrucker & Burdock, 2006). Licorice is widely used to moderate the effects (detoxification) of other herbs (Wang et al., 2013) in TCM and is often combined with *Fructus Psoraleae*. However, the classical TCM book, the *Ben Cao Qiu Zhen*, states that “*Fructus Psoraleae* is incompatible with Licorice”; in other words, *Fructus Psoraleae* should not be co-administered with Licorice. However, the mechanism underlying this incompatibility has not been identified.

The primary chemical ingredient of licorice is glycyrrhizin (GZ), which can be transformed to glycyrrhetinic acid (glycyrrhetic acid, GA) by intestinal bacteria following the oral intake of licorice in humans (Hattori et al., 1983; Hattori et al., 1985). GA is absorbed in blood as the active metabolite of licorice. GA inhibited the activities of cytochrome P450 isozymes, such as CYP2C9, CYP2C19 and CYP3A4 (Li et al., 2010; Liu et al., 2011), which are involved in the metabolic detoxification of BAK. We hypothesized that GA may increase the toxicity of BAK by inhibiting its detoxification enzymes CYP450s. In this study, the effect of concomitant GA administration on BAK-induced nephrotoxicity was investigated, and the metabolic interaction between BAK and GA was further studied *in vitro* and *in vivo*.

## Materials and Methods

### Drugs and reagents

BAK (Lot # zl131208659, purity ≥99% by HPLC) was purchased from Nanjing Zelang Medical Technology Co., LTD (Nanjing, China). GA (Lot # 110723-200612, purity ≥98% by HPLC), aristolochic acid A (purity: 99%), tanshinone II A (purity: 99%, internal standard for HPLC analysis), propranolol hydrochloride (internal standard) and midazolam were obtained from the National Institutes for Food and Drug Control (Beijing, China).

ABT (Lot# 100M4105, purity: 98%) and NADPH was purchased from Sigma; the following specific substrates and corresponding metabolites of CYP isoforms, phenacetin/acetaminophen (CYP1A2), tolbutamide/4-hydroxyl tolbutamide (CYP2C9), mephenytoin/4-hydroxyl mephenytoin (CYP2C19), dextromethorphan/dextrorphan (CYP2D6) and the midazolam metabolite 1-hydroxyl midazolam (CYP3A4), were products of Sigma; pooled HLMs (20 g/L, Lot # 7MMC011) were purchased from iPhase Pharmaceutical Services (Beijing, China); D/F12 (DMEM:F12 = 1:1) medium was purchased from Merck Millipore Beijing Skywing (Beijing, China); methanol and acetonitrile (HPLC-grade) were products of Fisher.

The liver and kidney toxicity detection kits, including alanine aminotransferase (ALT), asparagine acid transaminase (AST), urea nitrogen (BUN), creatinine (Cr) and beta-N-acetyl amino glycosidase enzyme (NAG) kits, were the products of Nanjing Jiancheng Bioengineering institute (Nanjing, China). The kidney injury molecule 1 (Kim-1) ELISA kit was purchased from Wuhan Boster Biological Engineering Co., LTD (Wuhan, China).

### Animals

Male Sprague–Dawley rats (weight ∼200 g) were obtained from the Department of Laboratory Animal Science at the Peking University Health Science Center. They were fed standard laboratory chow and had ad libitum access to water. The animal experiments were conducted according to a protocol approved by the Institutional Animal Care and Use Committee of Peking University (No.LA2014241), which complied with the guidelines of the Association for Assessment and Accreditation of Laboratory Animal Care International (AAALAC).

### Cell cultures

HK-2 cells obtained from Peking Union Medical College were maintained in D/F12 medium containing 10% fetal bovine serum, 2 mM glutamine and 1 × 10^5^ U/L penicillin and streptomycin at 37 °C in a 5% CO_2_ atmosphere.

### Cell viability

Six groups were tested in parallel to determine the effect of GA on BAK-induced cytotoxicity in HK-2 cells: the blank control group, vehicle control group (0.5% DMSO), BAK group (30, 45, 60, 75, 90 µM), BAK plus GA group (30 + 100, 45 + 100, 60 + 100, 75 + 100, 90 + 100 µM), BAK (30, 45, 60, 75, 90 µM) with HLMs (0.16 g/L) group, and BAK plus GA (30 + 100, 45 + 100, 60 + 100, 75 + 100, 90 + 100 µM) with HLMs (0.16 g/L) group.

The samples containing HLMs were prepared in an ice bath, and three replicates were tested for each dose. In brief, HK-2 cells (100 µL) were seeded at 3 × 10^5^cells/mL in 96-well plates. After 12 h, 200 µL of the test compound was added. For the BAK and GA with HLMs group, GA was pre-incubated with NADPH in HLMs for 30 min at 37 °C and then mixed with BAK; for the other groups, all tested compounds except NADPH were mixed, and NADPH was then added to start the metabolic reaction. After 4 h of culture, 100 µL of 0.5 g/L MTT was added, and the cells were cultured for 4 h. Subsequently, 150 µL of 4% isopropanol was added to cells for 15 min at 37 °C, and the absorbance at 570 nm was then read by a microplate reader (Multiskan MK3, Thermo, USA).

The relative cell viability (%) was calculated as follows: Mean Absorbance of Sample/Mean Absorbance of Vehicle Control × 100%.

### Metabolic elimination of BAK in HLMs

The effect of GA on the metabolism of BAK was evaluated by simultaneously determining the BAK and GA contents using HPLC after incubation with HLMs.

Four groups with three replicates each were tested: the control group, 0 time control group (enzyme inactivation group), BAK group (10 µM), and BAK plus GA co-incubation group (GA at 2.5, 12.5, 62.5, 93.75, and 125 µM). The HLM mixtures were prepared on ice.

For the (BAK + GA) group, GA was pre-incubated with NADPH in HLMs at 37 °C for 30 min and then incubated with BAK at 37 °C for 5 min. The reaction system included 0.5 g/L HLMs, 1 mM NADPH, and 10 µM BAK. At the end of the incubation (0 or 30 min), 200 µL of methanol-acetonitrile (1:1, V:V) solution containing tanshinone II A (internal standard) was added to terminate the reaction. The mixtures were then vortexed for 2 min followed by centrifugation at 14,500 rpm for 10 min. A 10 µL aliquot of the supernatant was then injected for HPLC analysis.

The inhibition of BAK metabolism by GA was assessed using the following formula: Percent inhibition (%) = (*R*_*GA*_ − *R*_*Contr*_)∕(1 − *R*_*Contr*_) × 100%, where R_*GA*_ is the percentage of BAK remaining in the BAK + GA group; R_*Contr*_ is the percentage of BAK remaining in the BAK group.

### Inhibition of CYP activities by GA

The effect of GA on the activities of CYP isoforms (CYP1A2, CYP2C9, CYP2C19, CYP2D6 and CYP3A4) was investigated in HLMs.

The following three groups were examined: the 0-time control group, HLMs with substrates group, and HLMs with substrates and various doses of GA (0.10, 1.00, 6.25, 31.25, 100.0, 156.2, 234.4, 312.5, and 1,000 µM) group. The samples containing HLMs were prepared in an ice bath, and three replicates of each dose were tested.

For the GA group, GA was pre-incubated with NADPH in HLMs at 37 °C for 30 min and then mixed with substrates, which were pre-incubated at 37 °C for 5 min. The reaction system consisted of 0.5 g/L HLMs, 1 mM NADPH, 25 µM phenacetin (CYP1A2 substrate), 25 µM tolbutamide (CYP2C9 substrate), 25 µM mephenytoin (CYP2C19 substrate), 10 µM dextromethorphan (CYP2D6 substrate) and 10 µM midazolam (CYP3A4 substrate). After incubation at 37 °C for 30 min, 200 µL of methanol containing 100 µg/L propranolol hydrochloride was added to terminate the reaction. The samples were then treated, and the corresponding substrate metabolites of CYP isoforms were detected according to a previously described method (Shen et al., 2013).

The relative activities of CYP isoforms were calculated as follows: E_*rel*_(%) = ci_(*n*)_ /ci_(0)_  × 100%, where ci_(*n*)_ is the relative amount of substrate metabolite in the GA group; ci_(0)_ is the relative amount of substrate metabolite in the control group.

The IC_50_ of GA for the CYP isoforms was calculated using the Origin 7.0 software and the Sigma-plot method.

### *In vivo* study

The SD rats were randomly divided into eight groups (*n* = 5 in each group) as follows: Saline control, Vehicle control (15% PEG 400 + 10% Tween 80), Nephrotoxicity positive control (aristolochic acid A, 70 mg/kg), ABT (100 mg/kg), GA (100 mg/kg), BAK (200 mg/kg), BAK plus ABT (200 + 100 mg/kg), and BAK plus GA (200 + 100 mg/kg). ABT, a broad-spectrum nonspecific CYP inhibitor, was selected as a positive control inhibitor of CYP enzymes. Aristolochic acid A (AA I) was selected as the nephrotoxicity positive control in the study.

The human average daily dose of *Fructus Psoraleae* is 6∼10 g, corresponding to 180–300 mg of BAK. The dose of BAK at 200 mg/kg in rats is slightly higher than the estimated human therapeutic dose. A single dose of GA for the volunteers is 150 mg in pharmacokinetics study (Zhao et al., 2008). The dose of GA at 100 mg/kg in rats is slightly higher than that of the therapeutic dose.

### Measurement of Hepatic and renal functional markers

The blood samples were taken 24 h after the administration of a single oral dose in each group. The samples were promptly centrifuged, and the biochemical parameters, including the levels of ALT, AST, BUN, Cr, and NAG, were then measured. The level of Kim-1 was measured in collected urine samples according to the kit instructions.

### Toxicokinetics and tissue exposure of BAK

After the administration of a single oral dose, blood samples were collected from the rats in three groups (BAK, BAK plus ABT, and BAK plus GA) at the following time points: 0.08, 0.25, 0.5, 1, 2, 4, 8, 12, and 24 h. The plasma samples were separated by centrifugation at 4000 rpm for 15 min. The liver and kidney tissue were also collected from individual rats 24 h after dosing, weighed and then homogenized in saline solution (0.6 g wet weight/mL) on ice. Subsequently, 10 µL of internal standard solution (tanshinone II A, 10 µg/mL) and 0.3 mL of acetonitrile were added to 0.1 mL of plasma or homogenates, and centrifuged at 13,300 rpm for 15 min after 2 min of vortexing. The supernatant was reconstituted in 100 µL of mobile phase after drying under 40 °C nitrogen, the mixture then was vortexed for 5 min and centrifuged at 13,300 rpm for 15 min. Finally, 20 µL of supernatant was injected for HPLC analysis.

### Analytical procedure

The amount of BAK and GA in the HLM mixtures was measured based on a previously developed HPLC method (Jiao et al., 2011).

The amount of BAK in the rat plasma or tissue samples was quantified using an Agilent 1260 HPLC system equipped with a Phenomenex C18 column (250 mm × 4.6 mm, 1.8 µm). The mobile phase consisted of methanol and water with 0.1% formic acid (89:11, v/v) and was supplied at a flow rate of 0.8 mL/min. The detection wavelength was set to 262 nm. The method validation data exhibited good linearity (*r* > 0.996) in the concentration range of 0.1∼10 μM. The recovery was >93%. The RSDs of intra-day and inter-day measurements were less than 5.9% and 7.9%, respectively, for *in vitro* samples; and less than 15% for *in vivo* samples.

### Statistical analysis

The results are expressed as the mean ± SD ($\bar {x}\hspace*{1em}\pm \hspace*{1em}$s). The statistical analysis was performed using the *SPSS 16.0* software. All data were statistically analyzed with a one-way analysis of variance (ANOVA), followed by a post-hoc test for individual group comparisons (Fisher PLSD test). Differences with *p* < 0.05 were considered significant.

## Results

### Effect of GA on BAK induced HK-2 cytotoxicity

The cytotoxicity was assessed using an MTT assay. In present study, 100 µM of GA was applied to observe an obvious effect on the toxicity of BAK. GA at a concentration of 100 µM or HLMs (0.16 g/L) did not affect HK-2 cell survival.

The cell viability data are shown in Table 1. As expected, the HK-2 cell viability significantly decreased in a dose-dependent manner after 4 h of exposure to BAK. The cell viabilities were significantly increased in the BAK/HLMs group, whereas the cell viabilities of the (BAK + GA)/HLMs group were significantly lower than those in the corresponding BAK/HLMs group (*p* < 0.01). These results suggest that GA attenuated the metabolic detoxification of BAK in HLMs.

### Inhibitory effect of GA on BAK metabolism

To investigate the effect of GA on the metabolism of BAK, BAK was co-incubated with GA in HLMs, and the percentage of BAK remaining at the end of incubation was measured. As shown in Table 2, the elimination of BAK in the (BAK + GA) group was significantly inhibited by 23%, 44%, 78% and 92% with different GA concentrations when compared with the BAK alone group. GA inhibited the metabolic clearance of BAK in a dose-dependent manner, and significant inhibition was observed at concentrations above 12.5 µM.

The relative content of GA (with time 0 as a control) did not significantly change after incubation with BAK for 30 min, which suggested that BAK (10 µM) did not markedly affect the metabolism of GA in HLMs (Table 2).

### Inhibitory effect of GA on CYP activities

**Figure 1:**
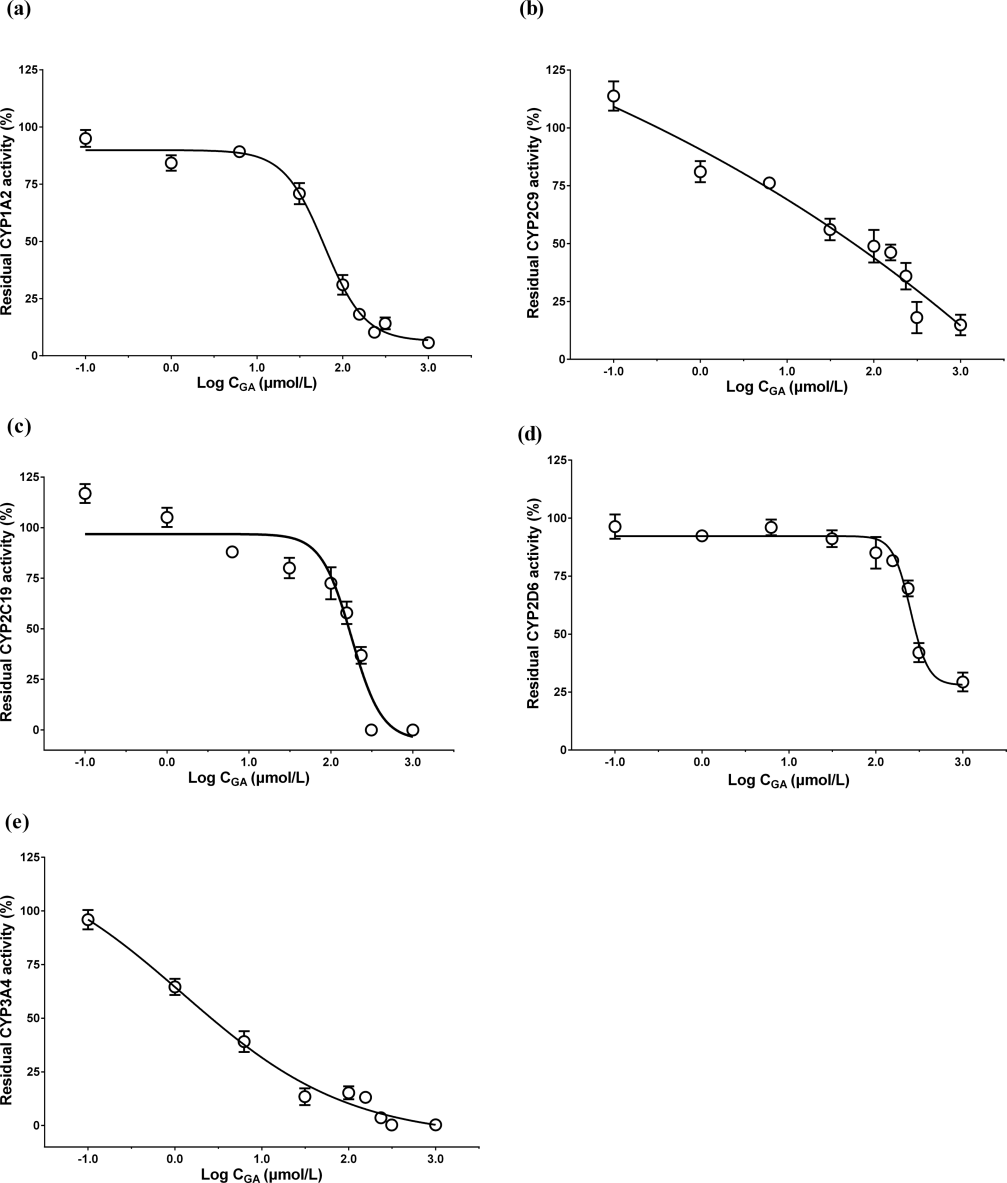
Sigma plots of the residual (A) CYP1A2, (B) CYP2C9, (C) CYP2C19, (D) CYP2D6, and (E) CYP3A4 activities vs. the logarithm of the concentration of GA. The relative activities of CYP isoforms after incubated with various concentration of GA for 30 min in HLMs. Data are expressed as mean ± SD (*n* = 3).

The effect of GA on the activities of CYP isoforms was examined by detecting the corresponding substrate metabolites using HPLC-MS/MS. The IC_50_ values were obtained from the sigma plots of the residual CYP activities *vs*. the logarithm of the concentration of GA (Fig. 1). As shown in Table 3, GA strongly inhibited CYP3A4, with an IC_50_ value of 1.53 µM. It also weakly inhibited CYP2C9 and CYP1A2.

**Table 1:** Effects of BAK and GA (100 µM) on HK-2 cell viability (4 h).

BAK (µM)	HK-2 Cell viability (%)		
	BAK	BAK/HLMs	(BAK + GA)/HLMs
**0**	100.00 ± 7.92	100.00 ± 9.59	100.00 ± 3.84
**30**	97.72 ± 10.68	103.77 ± 4.32	98.92 ± 3.55
**45**	84.59 ± 13.79	104.96 ± 9.39	98.72 ± 3.26
**60**	77.52 ± 4.69	100.98 ± 9.76	73.85 ± 5.38^**^
**75**	55.04 ± 11.33	109.38 ± 3.19	70.68 ± 8.56^**^
**90**	46.29 ± 12.46	92.53 ± 2.07	48.91 ± 4.40^**^

**Notes.**

Data are expressed as mean ± SD (*n* = 3).

** *p* < 0.01 compared with the same concentration of BAK with HLMs group.

### Effect of GA on toxicokinetics of BAK

**Figure 2:**
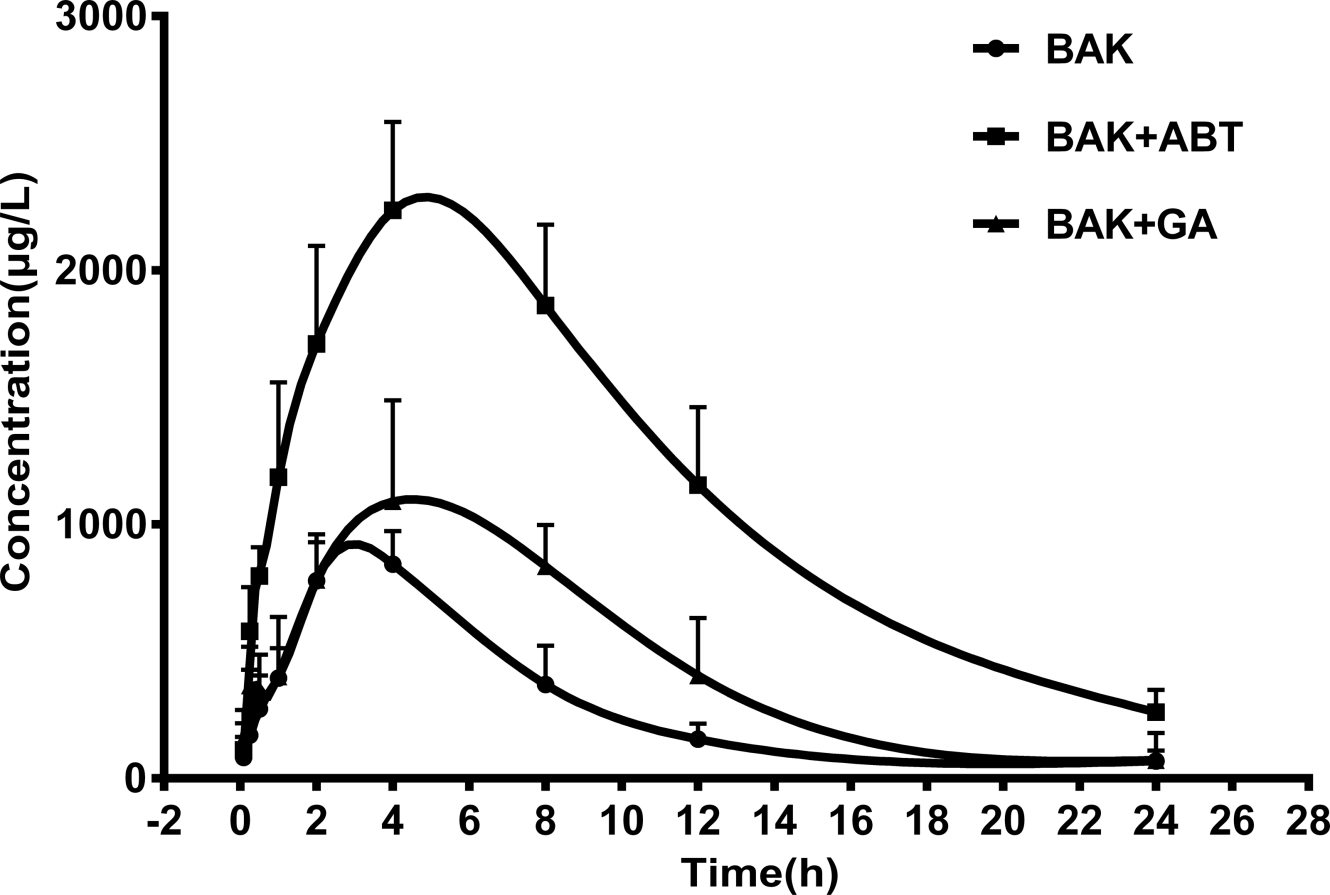
The mean plasma concentration–time profiles of BAK in rats after a single oral administration of BAK (200 mg/kg), BAK + ABT (200 + 100 mg/kg) , and BAK + GA (200 + 100 mg/kg). Data are expressed as mean ± SD (*n* = 5).

The time courses of mean BAK plasma concentrations were shown in Fig. 2. The toxicokinetic parameters were calculated using the non-compartmental model of DAS2.0 software, and the results are shown in Table 4. Compared with BAK alone, the plasma concentration area under curve up to 24 h (AUC_0−24_) and the maximum plasma concentration (Cmax) of BAK remarkably increased in the presence of ABT (*p* < 0.01). However, the plasma clearance (CL) declined significantly (*p* < 0.01). These results indicated that ABT inhibits the CYP-mediated metabolism of BAK, consequently both enhanced and extended the internal exposure of rats to BAK prototype.

**Table 2:** Inhibitory effect of GA on BAK metabolism in HLMs for 30 min.

GA concentration (µM)	Remaining percentage of BAK (%)	Remaining percentage of GA (%)	Inhibition of BAK by GA (%)
**0**	63.49 ± 6.05	–	–
**2.5**	63.55 ± 8.99	99.43 ± 28.88	−2.45 ± 32.33
**12.5**	71.60 ± 6.40	99.37 ± 9.39	22.72 ± 4.55
**62.5**	80.10 ± 6.81^*^	100.7 ± 12.33	43.85 ± 22.41
**93.8**	90.44 ± 15.33^*^	98.95 ± 3.80	77.87 ± 37.06
**125.0**	97.24 ± 1.46^**^	107.8 ± 7.54	92.00 ± 4.64

**Notes.**

Data are expressed as mean ± SD (*n* = 3).

* *p* < 0.05.

** *p* < 0.01, compared with the BAK group.

Similar to the effect of ABT, the combination of BAK and GA increased the AUC_0−24_ (*p* < 0.05), compared with BAK alone; whereas the CL declined (*p* < 0.05). The results showed that GA also inhibited the metabolism of BAK, but the extent of inhibition was weaker than that by ABT, which suggests that GA also might reduce the metabolism of BAK by inhibiting P450 enzymes, and slow down the elimination of BAK prototype.

### Effect of GA on BAK exposure in rat liver and kidney

**Figure 3:**
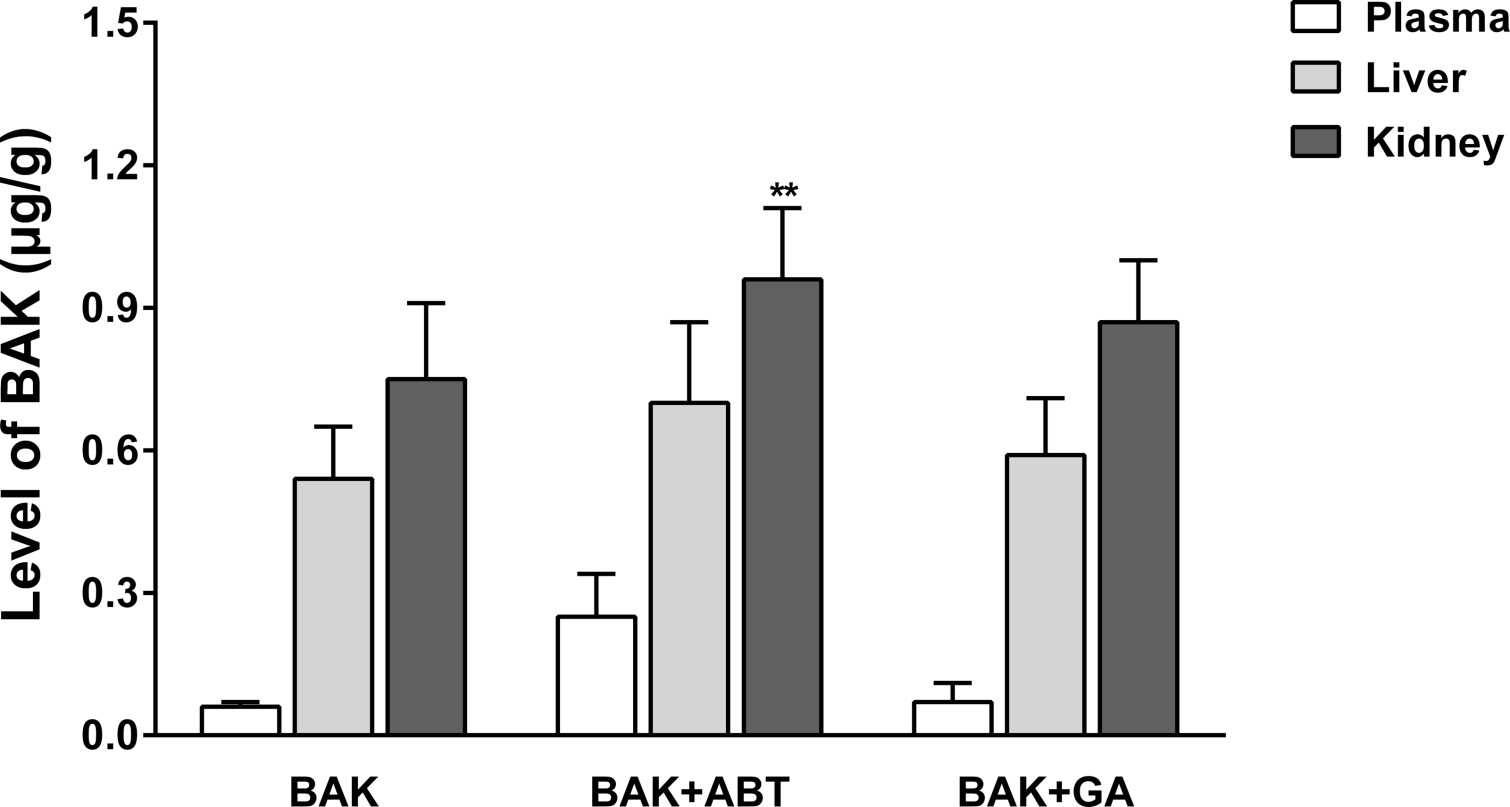
Effect of GA and ABT on the exposure of the rat liver and kidney to BAK. Data are expressed as mean ± SD (*n* = 5). **p < 0.01, compared with the BAK group.

The BAK exposure in liver and kidney tissues were measured 24 h after administration. As shown in Fig. 3, the levels of BAK descended in the following order: C _*kidney*_ > C _*liver*_ > C _*plasma*_. This result indicates that BAK likely accumulates in the liver and kidney, and that the kidney contained slightly more BAK than the liver (approximately 1.37- to 1.54-fold). Compared with the BAK group, the level of BAK in kidney in the presence of ABT increased by 27% (*p* < 0.01); upon the co-administration of the GA, increased by 15%, while this increment was not significant (*p* > 0.05).

**Table 3:** IC_50_ of CYP isoforms regulated by GA.

CYP isoforms	CYP1A2	CYP2C9	CYP2C19	CYP2D6	CYP3A4
IC_50_(µmol/L)	61.06 ± 1.13	26.46 ± 3.08	175.19 ± 1.26	263.01 ± 1.05	1.53 ± 2.25

**Notes.**

Data are expressed as mean ± SD (*n* = 3).

### Effects of GA and ABT on functional indicators of liver and kidney

The biochemical markers (ALT and AST content) did not significantly differ between the treatment groups and the vehicle control group (*p* > 0.05, Table S1). These results suggest that a single oral administration of high-dose BAK was not toxic to the rat liver.

**Figure 4:**
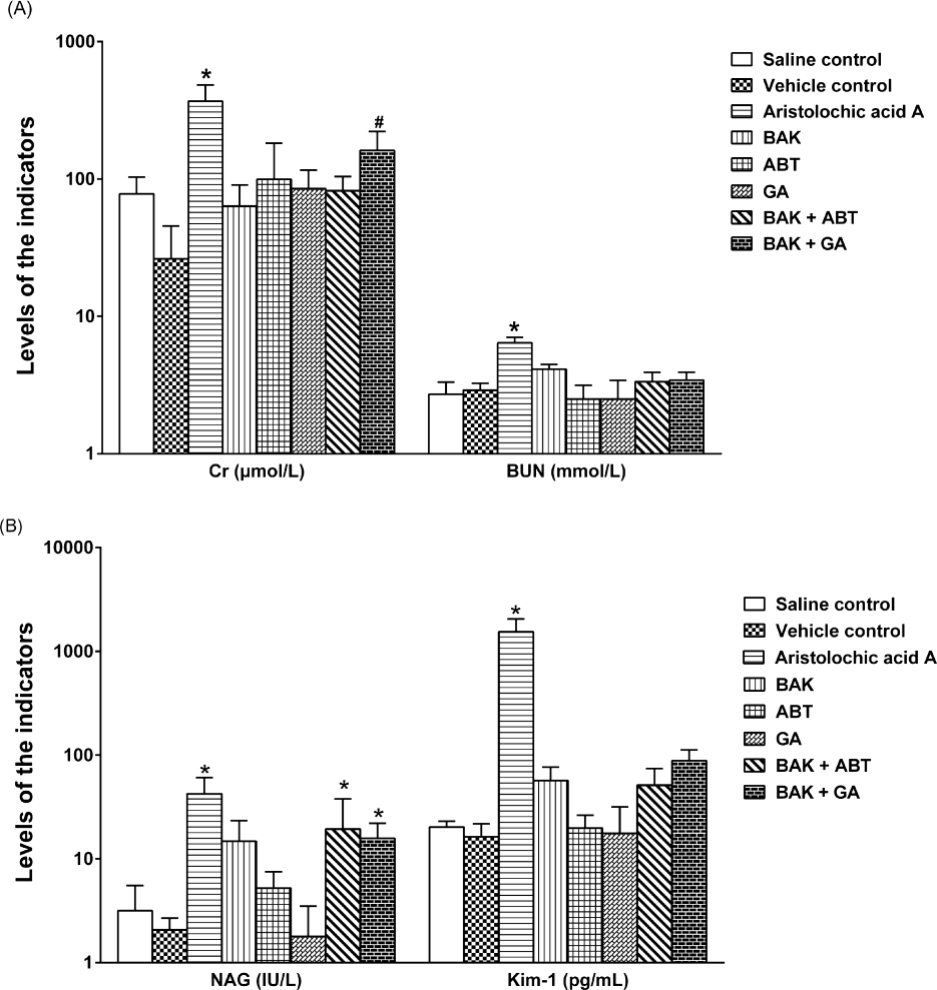
Effects on renal function in rats after a single oral dose. Data are expressed as mean ± SD (*n* = 5). *p < 0.05, compared with the vehicle control; # *p* < 0.05, compared with the BAK group.

As shown in Fig. 4, the four indicators (BUN, Cr, NAG and Kim-1) that reflect renal function significantly increased in the nephrotoxicity positive control group (AA I 70 mg/kg, *p* < 0.05), suggesting that the detection method and experimental results were reliable. These indicators did not significantly increase in the vehicle control group (*p* > 0.05) compared with the saline control, indicating that the solvents used in this experiment were not toxic to the kidney.

**Table 4:** Toxicokinetic parameters of BAK after a single oral administration.

Toxicokinetic parameters	BAK	BAK + ABT	BAK + GA
	(200 mg/kg)	(200 + 100 mg/kg)	(200 + 100 mg/kg)
AUC_0−24_ (h µg/L)	7,264 ± 2,141	28,841 ± 8,967^**^	11,978 ± 2,450^*^
*t*_1∕2_(h)	4.71 ± 1.56	7.20 ± 1.43	4.92 ± 1.26
*C*max (µg/L)	887.4 ± 280.6	2,416 ± 460.4^**^	1,090 ± 397.9
*T*max (h)	3.60 ± 0.89	4.80 ± 1.79	4.00 ± 0.86
CL (L/h/kg)	28.20 ± 7.91	6.77 ± 1.86^**^	16.50 ± 3.38^*^
MRT (h)	6.79 ± 0.43	8.19 ± 1.01	7.63 ± 0.96
V (L/kg)	184.2 ± 58.6	69.53 ± 18.02^*^	116.5 ± 35.3

**Notes.**

Data are expressed as mean ± SD (*n* = 5).

* *p* < 0.05.

** *p* < 0.01, compared with the BAK group.

Compared with the vehicle control group, the BAK group only slightly elevated (*p* > 0.05), while the (BAK + ABT) and (BAK + GA) groups both significantly elevated (*p* < 0.05) the level of NAG, suggesting that a single oral dose of BAK with GA or with ABT might damage the rat kidney. The level of BUN and Kim-1 did not significantly change (*p* > 0.05). Compared with the BAK group, the (BAK + GA) group significantly increased (*p* < 0.05) the Cr level. These results were consistent with the above-described significant inhibition of the CYP-mediated metabolism of BAK by GA or ABT, and consequently raised the level of BAK prototype in the kidney.

## Discussion

The cytotoxicity of BAK was reduced in the presence of HLMs, suggesting the metabolic detoxification of BAK by the enzymes in HLMs. Only at the high dose of BAK, the toxicity was observed due to the excess amount of BAK over the metabolic capacity of HLMs.

Licorice is commonly utilized as a medicinal herb and food supplement. In TCM, licorice is often used to moderate the characteristics of other herbs in a prescription, which is interpreted as detoxification (Qiao et al., 2012). For instance, the active metabolite of licorice, GA, reduces mesaconitine-induced toxicity (Sun et al., 2014). However, an ancient TCM book indicates that *Fructus Psoraleae* cannot be used with licorice, but the mechanism underlying this incompatibility is not known. In the present study, we provided preliminary evidence showing that these herbs interacted to inhibit CYPs, constituting the basis of this incompatibility.

CYPs are the major enzymes involved in drug metabolism. In terms of toxicity, the outcome is either metabolic detoxification or activation (Guengerich, 2008). CYP isozymes can be inhibited or induced by drugs, resulting in drug-drug interactions that can cause adverse reactions or reduce the effectiveness of therapies (Baillie, 2007; Ioannides, 2008; Choi, Chin & Kim, 2011). As the active metabolite of the main component in licorice, GA has been shown to modulate various CYP isoforms (Li et al., 2010; Liu et al., 2011), suggesting its potential to cause metabolic interactions.

Our results showed that GA inhibited the activities of CYP1A2, CYP2C9 and CYP3A4 in HLMs, and the IC_50_ values of GA for CYP2C9, CYP3A4, and CYP2D6 were similar to those reported in other studies (Li et al., 2010; Liu et al., 2011). The results indicated that GA strongly inhibited CYP3A4 and weakly inhibited CYP2C9 and CYP1A2. We previously found (Li et al., 2012) that BAK was metabolized by multiple enzymes, namely four CYP isoforms (CYP1A2, CYP2C9, CYP2C19 and CYP3A4) that are involved in the metabolism of BAK in HLMs. Hence, GA inhibited three CYP isoforms, which mediated the metabolism of BAK. Our data revealed that GA affected the metabolism of BAK in a dose-dependent manner, which was consistent with the observed inhibition of CYP isoform activities. These results suggest that the inhibition of CYP isoforms might be at least partially involved in the mechanism underlying the incompatibility of licorice with *Fructus Psoraleae*.

The toxicokinetic study of BAK confirmed that GA inhibited BAK metabolism *in vivo*. When co-administered with GA, the plasma concentration AUC of BAK was higher than the same dose of BAK alone; the plasma clearance was declined. These results indicated that GA inhibited the metabolism of BAK, suppressed the elimination of BAK, and therefore enhanced and extended the internal exposure of BAK prototype in rats. However, the extent of inhibition was weaker than that by ABT, a well-known broad-spectrum CYP inhibitor. Furthermore, the exposure of the kidney to BAK slightly increased when BAK was used in combination with GA or in the presence of ABT; consequently, the deliverable amount of BAK might accumulate in target tissue.

NAG is considered a sensitive indicator that can reflect early renal tubular injury and kidney damage (Waring & Moonie, 2011). Compared with the vehicle control, the level of NAG was significantly increased in both the (BAK + GA) and (BAK + ABT) groups (*p* < 0.05). The results revealed that renal toxicity in rats directly correlated with the exposure of the kidney to BAK.

CYP isoforms play a key role in reducing BAK-induced nephrotoxicity. The use of herbal medicines is currently increasing worldwide. Specifically, a growing number of patients take herbal medicines with other drugs, which increases the risk of herb-drug or herb-herb interactions (Gouws et al., 2012). BAK may be nephrotoxic if co-administered with drugs or herbal medicines that inhibit CYPs, such as GA. Licorice is often regarded as an antidote. However, in the case of BAK, it might promote toxicity, which contradicts the existing knowledge. The risk of herb-herb interactions and the clinical significance of these two herbs warrant further study. Moreover, a large number of consumers consume licorice as food, which may alter the efficacy of drugs or herbal medicines metabolized by CYP isoforms and are likely to result in adverse reactions.

## Conclusion

*In vitro* studies demonstrated that GA enhanced BAK-induced cytotoxicity by inhibiting CYP metabolism. *In vivo* investigations found that a single oral concomitant dose of BAK and GA increased the internal exposure of rats to BAK and enhanced renal toxicity. The present study demonstrated that GA inhibits CYP isoforms and subsequently might increase the nephrotoxicity of BAK, which may underlie one of the possible mechanisms responsible for the incompatibility between licorice and *Fructus Psoraleae*.

##  Supplemental Information

10.7717/peerj.2723/supp-1 Supplemental Information 1 NMR profiles for identification of BAK

10.7717/peerj.2723/supp-2 Table S1 Effects on liver function in rats after a single oral administration

10.7717/peerj.2723/supp-3 Supplemental Information 2 HK-2 Cell viability

10.7717/peerj.2723/supp-4 Supplemental Information 3 Inhibitory effect of GA on BAK metabolism in HLMs for 30 min

10.7717/peerj.2723/supp-5 Supplemental Information 4 Inhibitory effect of GA on CYP activities

10.7717/peerj.2723/supp-6 Data S1 *In vivo* data

## Additional Information and Declarations

### Data Availability

The following information was supplied regarding data availability: The raw data has been supplied as a Supplemental File.

## References

[ref-1] Adhikari S, Joshi R, Patro BS, Ghanty TK, Chintalwar GJ, Sharma A, Chattopadhyay S, Mukherjee T (2003). Antioxidant activity of bakuchiol: experimental evidences and theoretical treatments on the possible involvement of the terpenoid chain. Chemical Research in Toxicology.

[ref-2] Baillie TA (2007). Metabolism and toxicity of drugs. Two decades of progress in industrial drug metabolism. Chemical Research in Toxicology.

[ref-3] Chaudhuri RK, Bojanowski K (2014). Bakuchiol: a retinol-like functional compound revealed by gene expression profiling and clinically proven to have anti-aging effects. International Journal of Cosmetic Science.

[ref-4] Chen Z, Jin K, Gao L, Lou G, Jin Y, Yu Y, Lou Y (2010). Anti-tumor effects of bakuchiol, an analogue of resveratrol, on human lung adenocarcinoma A549 cell line. European Journal of Pharmacology.

[ref-5] Cho H, Jun JY, Song EK, Kang KH, Baek HY, Ko YS, Kim YC (2001). Bakuchiol: a hepatoprotective compound of *Psoralea corylifolia* on tacrine-induced cytotoxicity in Hep G2 cells. Planta Medica.

[ref-6] Choi YH, Chin YW, Kim YG (2011). Herb-Drug interactions: focus on metabolic enzymes and transporters. Archives of Pharmacal Research.

[ref-7] Chopra B, Dhingra AK, Dhar KL (2013). *Psoralea corylifolia* L. (Buguchi)—Folklore to modem evidence: review. Fitoterapia.

[ref-8] Gouws C, Steyn D, Du Plessis L, Steenekamp J, Hamman JH (2012). Combination therapy of Western drugs and herbal medicines: recent advances in understanding interactions involving metabolism and efflux. Expert Opinion on Drug Metabolism & toxicology.

[ref-9] Guengerich FP (2008). Cytochrome P450 and chemical toxicology. Chemical Research in Toxicology.

[ref-10] Hattori M, Sakamoto T, Kobashi K, Namba T (1983). Metabolism of glycyrrhizin by human intestinal flora. Planta Medica.

[ref-11] Hattori M, Sakamoto T, Yamagishi T, Sakamoto K, Konishi K, Kobashi K, Namba T (1985). Metabolism of glycyrrhizin by human intestinal flora. 2 isolation and characterization of human intestinal bacteria capable of metabolizing glycyrrhizin and related-compounds. Chemical & Pharmaceutical Bulletin.

[ref-12] Ioannides C (2008). Cytochromes P450: role in the metabolism and toxicity of drugs and other xenobiotics.

[ref-13] Isbrucker RA, Burdock GA (2006). Risk and safety assessment on the consumption of Licorice root (Glycyrrhiza sp.), its extract and powder as a food ingredient, with emphasis on the pharmacology and toxicology of glycyrrhizin. Regulatory Toxicology and Pharmacology.

[ref-14] Iwamura J, Dohi T, Tanaka H, Odani T, Kubo M (1989). Cytotoxicity of corylifoliae fructus II. Cytotoxicity of bakuchiol and the analogues. Yakugaku Zasshi.

[ref-15] Jiang F, Zhou X-R, Wang Q, Zhang B-X (2010). Cytotoxic effect and mechanism of bakuchiol and bakuchiol combined with psoralen on HK-2 cell. Zhongguo Yaolixue Yu Dulixue Zazhi.

[ref-16] Jiao S-Y, Ai C-H, Li A-F, Li H, Wang Q (2011). *In vitro* inter-species comparison of metabolism and metabolic detoxification of bakuchiol in liver microsomes. Zhongguo Yaolixue Tongbao.

[ref-17] Li AF, Shen GL, Jiao SY, Li H, Wang Q (2012). Metabolic detoxification of bakuchiol is mediated by cytochrome P450 enzymes in human liver microsomes. Beijing Da Xue Xue Bao.

[ref-18] Li HY, Xu W, Su J, Zhang X, Hu LW, Zhang WD (2010). *In vitro* and *in vivo* inhibitory effects of glycyrrhetinic acid on cytochrome P450 3A activity. Pharmacology.

[ref-19] Liu L, Xiao J, Peng Z-H, Chen Y (2011). *In vitro* metabolism of glycyrrhetic acid by human cytochrome P450. Yao Xue Xue Bao = Acta Pharmaceutica Sinica.

[ref-20] Park EJ, Zhao YZ, Kim YC, Sohn DH (2005). Protective effect of (S)-bakuchiol from *Psoralea corylifolia* on rat liver injury *in vitro* and *in vivo*. Planta Medica.

[ref-21] Qiao CF, Han QB, Song JZ, Mo SF, Kong LD, Kung HF, Xu HX (2007). Chemical fingerprint and quantitative analysis of fructus psoraleae by high-performance liquid chromatography. Journal of Separation Science.

[ref-22] Qiao X, Ye M, Xiang C, Bo T, Yang WZ, Liu CF, Miao WJ, Guo DA (2012). Metabolic regulatory effects of licorice: a bile acid metabonomic study by liquid chromatography coupled with tandem mass spectrometry. Steroids.

[ref-23] Shen GL, Zhong YH, Yuan M, Zhuang XM, Li H (2013). Simultaneous quantitation of six cytochrome P450 enzyme probe metabolites by ultra-high performance liquid chromatography tandem mass spectrometry. Chinese Journal of Analytical Chemistry.

[ref-24] Sun B, Zhang M, Zhang Q, Ma K, Li H, Li F, Dong F, Yan X (2014). Metabonomics study of the effects of pretreatment with glycyrrhetinic acid on mesaconitine-induced toxicity in rats. Journal of Ethnopharmacology.

[ref-25] Wang X, Zhang H, Chen L, Shan L, Fan G, Gao X (2013). Liquorice, a unique “guide drug” of traditional Chinese medicine: a review of its role in drug interactions. Journal of Ethnopharmacology.

[ref-26] Waring WS, Moonie A (2011). Earlier recognition of nephrotoxicity using novel biomarkers of acute kidney injury. Clinical Toxicology.

[ref-27] Zhang YS (1981). Bu gu zhi fen dui xiao shu shen zang du hai zuo yong de yan jiu (A study on toxicity of bakuchiol to mice’s kidney (author’s translation)). Zhong Yao Tong Bao.

[ref-28] Zhao WJ, Wang BJ, Wei CM, Yuan GY, Bu FL, Guo RC (2008). Determination of glycyrrhetic acid in human plasma by HPLC-MS method and investigation of its pharmacokinetics. Journal of Clinical Pharmacy and Therapeutics.

