# Supplementary figures and images for: Glycyrrhetinic acid might increase the nephrotoxicity of bakuchiol by inhibiting cytochrome P450 isoenzymes
Source: PeerJ. 2016 Nov 22;4:e2723. doi: 10.7717/peerj.2723 (PMC5126668; doi:10.7717/peerj.2723)

A

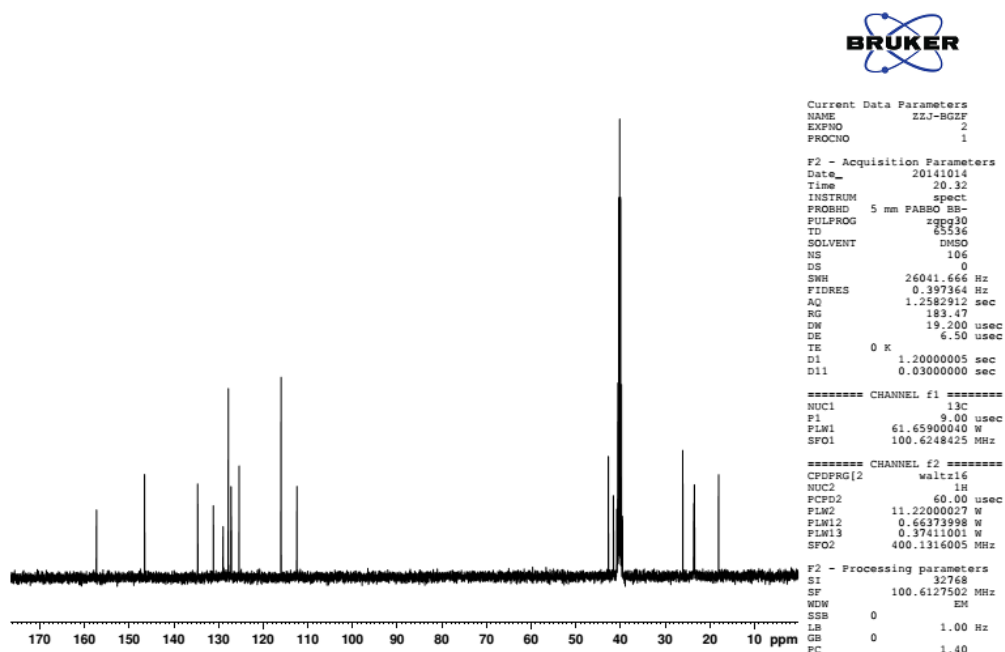

B

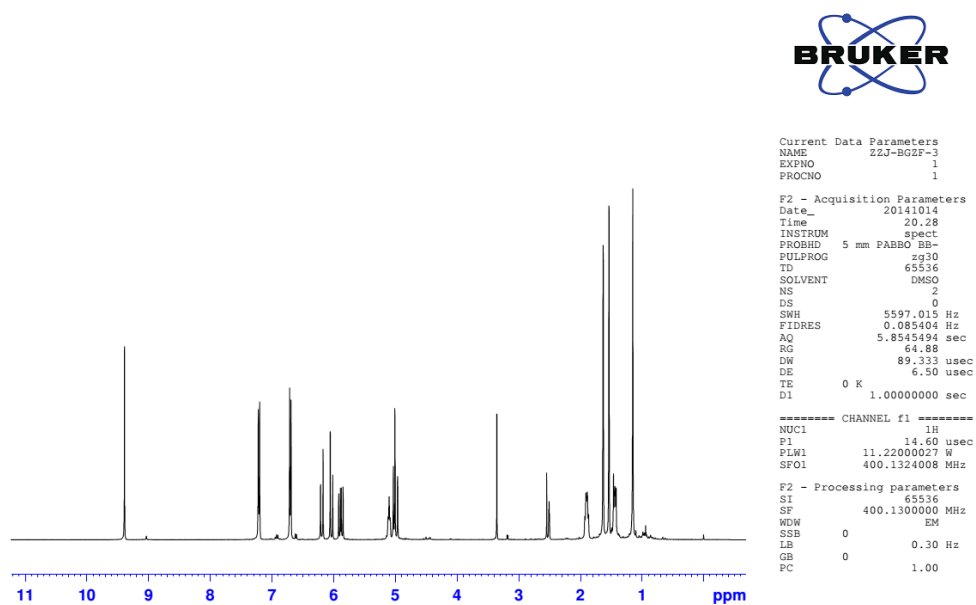

Fig. S1. Identification of bakuchiol (Lot # zl131208659). (A) C-NMR profile. (B) H-NMR profile.

Supplement: Supplemental Information 1 [file peerj-04-2723-s001.pdf]
