# Supplementary material for: Glycyrrhetinic acid might increase the nephrotoxicity of bakuchiol by inhibiting cytochrome P450 isoenzymes
Source: PeerJ. 2016 Nov 22;4:e2723. doi: 10.7717/peerj.2723 (PMC5126668; doi:10.7717/peerj.2723)
Supplement: Table S1 [file peerj-04-2723-s002.docx]

**Effects on liver function in rats after a single oral administration**

| Group (mg/kg) | ALT (IU/L) | AST (IU/L) |
| --- | --- | --- |
| Saline control | 47.41±3.50 | 28.33±19.97 |
| Vehicle control | 49.38±5.51 | 38.63±11.31 |
| Positive control (AA І, 70) | 41.91±3.40 | 29.08±8.01 |
| BAK 200 | 43.11±3.89 | 17.45±0.54 |
| ABT 100 | 50.09±4.57 | 38.044±9.99 |
| GA 100 | 40.50±8.61 | 28.16±9.53 |
| BAK + ABT (200+100) | 58.61±16.40 | 40.22±20.02 |
| BAK + GA (200+100) | 60.38±14.70 | 39.89±27.50 |

Data are expressed as mean ± SD（n=5）
